# Complex effects of atmospheric parameters on acute cardiovascular diseases and major cardiovascular risk factors: data from the Cardiometeorology^SM^ study

**DOI:** 10.1038/s41598-019-42830-6

**Published:** 2019-04-23

**Authors:** Nora Boussoussou, Melinda Boussoussou, Gergő Merész, Márton Rakovics, László Entz, Attila Nemes

**Affiliations:** 1 Semmelweis University, Department of Vascular Surgery, 68. Városmajor street, Budapest, 1122 Hungary; 2 Semmelweis University, Department of Behavioural Sciences, Budapest, Hungary; 3 Eötvös Loránd University, Department of Statistics, Budapest, Hungary

**Keywords:** Atmospheric science, Cardiology, Risk factors

## Abstract

Several studies have examined the cardiovascular effects of atmospheric parameters as separate factors; however, few have investigated atmospheric parameters’ joint effects. We aim to explore the joint effects of atmospheric parameters on acute cardiovascular diseases (ACVDs) and on major cardiovascular risk factors (CRFs). We correlated all ACVD admissions with major CRFs and local atmospheric conditions during a 5-year study period. A seasonal variation was detected in a higher incidence rate during cold atmospheric conditions. There were significant incidence relative ratios, including: 1.140 (95% CI [1.020, 1.283]) for daily temperature change (≥5 °C); 0.991 (95% CI [0.988, 0.994]) for average daily temperature; and 1.290 (95% CI [1.090, 1.599]) for the interaction of daily temperature change (≥5 °C) with humidity change (≥40%). We observed a significant association between the atmospheric parameters’ joint effects and hyperlipidaemia, diabetes, and previous ACVDs. Patients with diabetes had the highest significant incidence relative ratio at 2.429 (95% CI [1.088, 5.424]) for humidity-temperature interactions. Thus, the atmospheric parameters’ joint effects play an important role as minor CRFs. These unfavourable atmospheric situations are predicted to increase the number of ACVDs mainly. Our study may help to organize prevention strategies more effectively and to reduce cardiovascular risks.

## Introduction

There is substantial evidence that the health threat of global climate change is real, and it is a medical emergency. The Lancet Commission on Health and Climate Change has declared that the biggest health challenge in the 21^st^ century is climate change^1^. Unfavourable atmospheric situations caused by climate change are predicted to increase the number of acute cardiovascular diseases (ACVDs) mainly. ACVDs are already major public health issues, and, in the future, adverse atmospheric parameters can further increase this problem. Europe, North-East America, and North Asia are the most affected geographical regions in terms of extreme atmospheric parameters. Additionally, various epidemiological studies have shown that there is a seasonal variation in the incidence of ACVDs. A greater ACVD incidence, both during cold and warm temperatures, has been detected^2–8^. Few studies have shown an association between atmospheric pressure, humidity, wind, sunlight, and cardiovascular diseases (CVDs)^9–12^. Nevertheless, most studies have mentioned the aforementioned atmospheric parameters as separate factors.

The aim of our Cardiometeorology^SM^ study was to investigate the joint effects of atmospheric parameters on ACVD incidences and on major CRFs. To our knowledge, this is the first study to qualify the association between the joint effects of atmospheric parameters and major CRFs based on ACVD hospitalizations. In light of global climate change, it is essential to focus on atmospheric parameters, such as minor CRFs. According to the Fourth Assessment Report of the Intergovernmental Panel on Climate Change in 2007, extreme weather conditions and rapid, short-term changes in atmospheric conditions will become more and more frequent in the future^13^. Thus, a better understanding of atmospheric parameters can help establish new cardiovascular prevention strategies against them.

## Results

**Figure 1:**
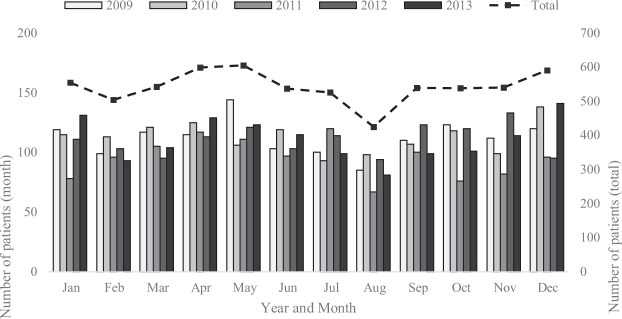
The monthly and aggregated number of patients for each month in all years. Monthly number of patients (left axis) and aggregated number of patients for each month in all years (right axis). Greyscale bars show number of patients for all months for years 2009–2013, measured on the left axis. The dashed line shows total number of patients by year, measured of the right axis.

In a 5-year period from 2009–2013, 6,499 patients were admitted to the Department of Vascular Surgery of Semmelweis University with a diagnosis of ACVD. The number of monthly ACVD hospitalizations and the aggregated number of patients for each month in every year are shown in Fig. 1. The seasonal admission for ACVDs is also shown. The aggregated number of hospitalizations was moderately stable throughout all years; however, there was an observable increase in the total monthly hospitalizations in the months of late spring and towards the end of the calendar year. The lowest number of hospitalizations could be observed in August. This attests to the presence of substantial seasonality in the data.

**Figure 2:**
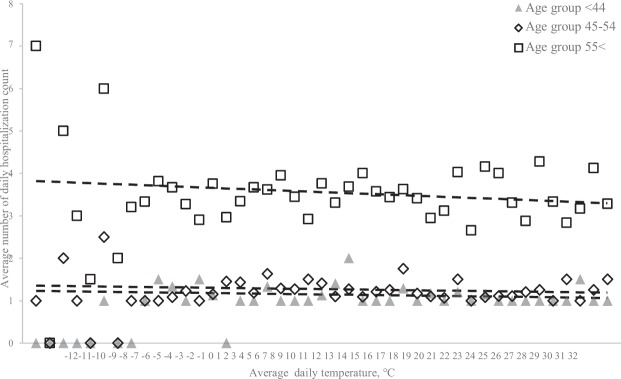
Average number of daily hospital admissions by average daily temperature, (°C). Markers show the average number for patient admissions by average daily temperature (°C) for each age group. Triangles are average counts for the age group <44, diamonds are for age group 45–54, squares are for age group 55<. Dashed lines show a downward sloping linear trend in average counts for higher average daily temperatures in all three groups.

As part of the descriptive analysis, the average daily hospitalisation count was plotted against the average daily atmospheric temperature by age group, showing a slight but consistent negative association, which appeared to be more dominant as age increased. In Fig. 2, linear lines represent the trendlines that were fitted for each age group.

**Figure 3:**
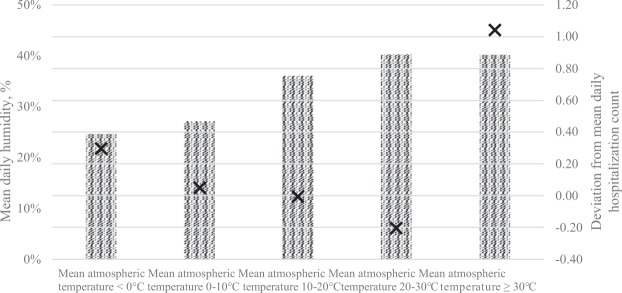
Humidity and average daily event counts by categories of atmospheric temperature. Relationship of humidity and average daily event counts with different categories of mean atmospheric temperature. Bars show average humidity by mean temperature categories, measured on the left axis. Cross markers show differences of average daily event counts by categories of atmospheric temperature from the grand mean, measured on the right axis.

To evaluate the effect of atmospheric temperature on the daily hospitalization counts by exploring the potential interaction with other atmospheric parameters, the mean daily humidity was also described by estimating the average humidity and the standard deviation from the overall mean of daily event counts according to the mean daily temperature categories. Figure 3 confirms a negative association between the temperature and hospitalization counts; however, there were also yields of high humidity and high hospitalization counts on days with an average temperature of ≥30 °C, suggesting an interaction between these two factors.

**Figure 4:**
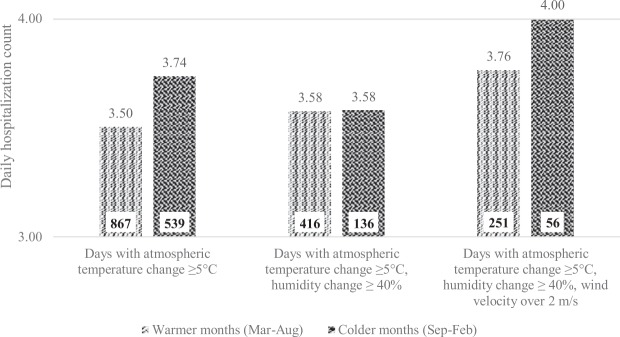
Average daily event counts and number of days with specific weather conditions by warmer and colder months of the year. Daily number of hospital admissions for different weather conditions by colder and warmer months. Lighter bars show average of daily counts for warmer months (March to August), darker bars show average of daily counts for colder months (September to February). Numbers at the base of bars show total counts for each category of weather conditions and months.

To further explore interactions between atmospheric parameters and seasonality, daily hospitalization counts were estimated according to categories of variation in temperature, humidity, and mean daily wind velocity according to colder and warmer months. Figure 4 shows that the interaction between the temperature and humidity changes yielded a differential effect in colder and warmer months, whereas adding wind velocity increased the mean daily hospitalization counts in both warmer and colder months.

To correct for these effects in both the mean and variation of atmospheric parameters, a multivariate regression approach was adapted to estimate the effects of atmospheric parameters on daily hospitalization counts. The Poisson-regression model was applied and used covariates as explanatory variables to capture the annual seasonality in the data. The results of the multivariate regression analyses are shown in Table 1. The daily mean and variation in atmospheric temperature were shown to have statistically significant associations with the daily ACVD hospitalizations; for example, a daily temperature change of ≥5 °C was associated with an average 14.0% increase in hospitalization counts, and a 1 °C decrease in average daily atmospheric temperature was associated with a modest but statistically significant increase of 0.9%.

**Table 1:** Relative risk estimates for atmospheric parameters from Poisson model to estimate daily event counts (n = 6′499).

Parameter*	Relative risk	CI 95% lower	CI 95% upper	p-value
**Daily atmospheric temperature change** ≥**5** °**C**	**1**.**140**	**1**.**020**	**1**.**283**	**0**.**021**
Daily atmospheric air pressure change ≥10 hPa	0.986	0.894	1.092	0.798
Daily humidity change ≥40%	0.798	0.643	0.950	0.051
**Average daily atmospheric temperature**, **°C**	**0**.**991**	**0**.**988**	**0**.**994**	<**0**.**001**
Average daily atmospheric pressure, hPa	1.001	0.999	1.004	0.285
Average daily humidity, %	0.999	0.997	1.001	0.589
Average daily wind velocity over 2 m/s	1.054	0.960	1.151	0.300
**Interaction of daily atmospheric temperature change** ≥**5 °C and humidity change** ≥**40%**	**1**.**290**	**1**.**090**	**1**.**599**	**0**.**034**
Interaction of daily atmospheric temperature change ≥5 °C and wind velocity ≥2 m/s	0.967	0.863	1.074	0.576

*Parameters are adjusted for seasonality. Bold rows indicate parameters significantly associated with daily event counts (p ≤ 0.05).

The term “daily” is used to refer to event counts and weather parameters observed over one calendar day. A 5 °C change was included as an indicator variable, with a value of 1 if the difference between the daytime maximum and minimum temperature exceeded 5 °C and a value of 0 otherwise. The adjustment for the variation in temperature attributable to the change from night-time to daytime was addressed implicitly when accounting for seasonality, as there is no general rule for the size of this effect, and there was only one measurement of weather pattern per day. The adjustment for the effect of seasonality on the index day was carried out by considering the changes in the observed variables from one day before and, additionally, from 365 days before. This implicates that the difference in the effect of the weather parameters on the hospitalization event counts across the whole year is similar throughout the years; therefore, even if there was a bias attributable to the 5 °C changes not being distributed evenly over the course of a year, the overall effect should be similar across a longer time horizon.

The interaction between a daily temperature change of ≥5 °C and a daily humidity change of ≥40% showed a positive association with daily hospitalization counts, increasing the daily number of hospitalizations by 29.0%. Table 2 shows further analyses that reveal consistent findings when fitting the same regression model for the subpopulations of hypertensive, diabetic, and hyperlipidaemic patients as well as patients with a history of previous ACVDs. The regression models revealed a significant association between atmospheric interactions, changes, and subpopulations, including hyperlipidaemia, diabetes, and previous ACVD history. The highest significant association that was detected between CRFs and the atmospheric parameters’ interactions was the association between the humidity-temperature interaction and diabetes. The effect of the humidity and temperature interactions was positively associated with the daily hospitalization counts on days with a temperature change of ≥5 °C and a humidity change of 40%; the mean number of hospitalizations increased by an average of 142.9%.

**Table 2:** Relative risk estimates of subpopulation with hypertensive, diabetic, hyperlipidemic patients and patients with history of previous CVD associated with atmospheric parameters, lagged by 2 days.

Parameter*	Relative risk	CI 95% lower	CI 95% upper	p-value
Relative risk estimates for atmospheric parameters from Poisson model to estimate daily event counts for the hypertensive subsample (n = 2,886)				
Daily atmospheric temperature change ≥5 °C	1.018	0.858	1.207	0.850
Daily atmospheric air pressure change ≥10 hPa	0.970	0.830	1.134	0.717
Daily humidity change ≥40%	0.826	0.609	1.121	0.222
Average daily atmospheric temperature, °C	0.996	0.990	1.002	0.185
**Average daily atmospheric pressure**, **hPa**	**1**.**006**	**1**.**000**	**1**.**012**	**0**.**036**
**Average daily humidity**, **%**	**0**.**992**	**0**.**989**	**0**.**996**	<**0**.**001**
Average daily wind velocity over 2 m/s	1.114	0.937	1.325	0.224
Interaction of daily atmospheric temperature change ≥5 °C and humidity change ≥40%	1.211	0.882	1.662	0.240
Interaction of daily atmospheric temperature change ≥5 °C and wind velocity ≥2 m/s	1.119	0.923	1.356	0.255
Relative risk estimates for atmospheric parameters from Poisson model to estimate daily event counts for the diabetic subsample (n = 1,074)				
**Daily atmospheric temperature change** ≥**5 °C**	**1**.**480**	**1**.**062**	**2**.**062**	**0**.**020**
Daily atmospheric air pressure change ≥10 hPa	0.975	0.765	1.244	0.850
**Daily humidity change** ≥**40%**	**0**.**351**	**0**.**159**	**0**.**773**	**0**.**009**
**Average daily atmospheric temperature**, **°C**	**0**.**987**	**0**.**978**	**0**.**997**	**0**.**009**
Average daily atmospheric pressure, hPa	0.999	0.991	1.008	0.898
**Average daily humidity**, **%**	**0**.**992**	**0**.**987**	**0**.**998**	**0**.**005**
**Average daily wind velocity over 2 m/s**	**1**.**693**	**1**.**214**	**2**.**360**	**0**.**002**
**Interaction of daily atmospheric temperature change** ≥**5 °C and humidity change** ≥**40%**	**2**.**429**	**1**.**088**	**5**.**424**	**0**.**030**
Interaction of daily atmospheric temperature change ≥5 °C and wind velocity ≥2 m/s	1.010	0.703	1.451	0.961
Relative risk estimates for atmospheric parameters from Poisson model to estimate daily event counts for the CVD subsample (n = 1,004)				
Daily atmospheric temperature change ≥5 °C	1.190	0.881	1.606	0.260
Daily atmospheric air pressure change ≥10 hPa	0.815	0.613	1.081	0.156
**Daily humidity change** ≥**40%**	**1**.**659**	**1**.**045**	**2**.**633**	**0**.**031**
Average daily atmospheric temperature, °C	0.997	0.987	1.007	0.524
Average daily atmospheric pressure, hPa	1.000	0.991	1.010	0.960
**Average daily humidity**, **%**	**0**.**993**	**0**.**988**	**0**.**999**	**0**.**021**
Average daily wind velocity over 2 m/s	1.038	0.756	1.425	0.829
**Interaction of daily atmospheric temperature change** ≥**5** °**C and humidity change** ≥**40%**	**0**.**511**	**0**.**314**	**0**.**830**	**0**.**007**
Interaction of daily atmospheric temperature change ≥5 °C and wind velocity ≥2 m/s	1.339	0.946	1.894	0.099
Relative risk estimates for atmospheric parameters from Poisson model to estimate daily event counts for the hyperlipidaemia subsample (n = 1,967)				
Daily atmospheric temperature change ≥5 °C	0.882	0.721	1.078	0.223
Daily atmospheric air pressure change ≥10 hPa	0.989	0.817	1.198	0.918
Daily humidity change ≥40%	0.788	0.533	1.166	0.236
Average daily atmospheric temperature, °C	0.999	0.992	1.006	0.753
**Average daily atmospheric pressure**, **hPa**	**1**.**008**	**1**.**002**	**1**.**015**	**0**.**015**
**Average daily humidity**, **%**	**0**.**991**	**0**.**987**	**0**.**995**	<**0**.**001**
Average daily wind velocity over 2 m/s	0.950	0.772	1.170	0.642
Interaction of daily atmospheric temperature change ≥5 °C and humidity change ≥40%	1.280	0.853	1.920	0.235
**Interaction of daily atmospheric temperature change** ≥**5** **°C and wind velocity** ≥**2** **m/s**	**1**.**317**	**1**.**046**	**1**.**658**	**0**.**019**

*Parameters are adjusted for seasonality. Bold rows indicate parameters significantly associated with daily event counts (p ≤ 0.05).

The effects of atmospheric parameters were also tested on the daily hospitalization counts of patients with different ACVDs: acute myocardial infarction (AMI), pulmonary embolism (PE), dissection of the aorta (AD), aortic aneurysm rupture (AAR), thromboembolism. Table 3 shows that daily temperature change of ≥5 °C and average daily temperature in the AMI group were the only significant results due to small sample sizes in the other groups, but there was no statistical evidence for differences of weather parameters across groups.

**Table 3:** Relative risk estimates for atmospheric parameters from Poisson model to estimate daily event counts of different AVCDs (AMI: n = 5,221; thromboembolism: n = 1,211; AAR, AD: n = 28; PE: n = 63).

Parameter*	Relative Risk				p-value for comparing estimates
	AMI	thrombo-embolism	AAR, AD	PE	
Daily atmospheric temperature change ≥5 °C	**1**.**190**	1.011	0.775	1.451	0.253
Daily atmospheric air pressure change ≥10 hPa	0.909	1.29	1.038	0.600	0.979
Daily humidity change ≥40%	0.845	0.621	0.001	1.109	0.433
Average daily atmospheric temperature, °C	**0**.**989**	0.996	0.974	1.011	0.680
Average daily atmospheric pressure, hPa	1.002	0.998	0.999	0.997	0.360
Average daily humidity, %	0.999	0.998	0.996	1.01	0.192
Average daily wind velocity over 2 m/s	1.018	1.167	0.677	3.486	0.183
Interaction of daily atmospheric temperature change ≥5 °C and humidity change ≥40%	1.224	1.678	NA	0.974	0.433
Interaction of daily atmospheric temperature change ≥5 °C and wind velocity ≥2 m/s	0.989	0.894	2.006	0.323	0.070

*Parameters are adjusted for seasonality. Bold values indicate parameters significantly associated with daily event counts (p ≤ 0.05). NA indicates that reliable parameter estimation failed due to small sample size.

## Discussion

The principal finding of this study shows that daily atmospheric parameter changes based on temperature, humidity, and wind interactions are associated with ACVDs, and that the relative risk varied by age and other major CRFs. To our knowledge, this is the first reported retrospective, observational, population-based study in Central-Europe to identify the association between the atmospheric parameters’ complex interactions and the ACVD hospital admissions with major CRFs. Moreover, the seasonality of cardiovascular morbidity is well known. Our results further illustrated a trend of a seasonal variation in the incidence of ACVDs; a higher hospitalisation rate during the cold season was also detected.

Cold temperature causes several pathophysiological changes that can act as a minor CRF. At low temperatures, upregulation of the sympathetic nervous system occurs. Cardiac contractility, frequency, the rate of relaxation, and the impulse conduction through the atrioventricular node also increase. As a result of the cold, the plasma catecholamine and vasopressin levels are increased, and the activity of the renin angiotensin system intensifies^14^. These mechanisms lead to an increase in blood pressure. Furthermore, the cold exerts negative effects on inflammation and haemostasis. The levels of C-reactive protein, Interleukin 6, P- and E-selectin, intercellular adhesion molecule 1, vascular cell adhesion molecule 1, and the levels of haemoglobin, haematocrit, red blood cells, white blood cell count, thrombocytes, plasminogen activator inhibitor type 1, α2-macroglobulin, factor VII, and fibrinogen are increased^15–18^. These elevated levels lead to an inflammatory and hypercoagulable status, with a higher risk of developing thromboembolism.

Moreover, during cold effects, the level of Endothelin 1 is increased while the endothelial nitrogen monoxide level is decreased^19^. In winter, higher levels of low-density lipoprotein cholesterol and lower levels of high-density lipoprotein cholesterol can be detected^20^. Exposure to the cold has also been shown to affect thyroid hormone levels, causing triiodothyronine and thyroxine levels to decrease and thyroid-stimulating hormone levels to rise^21^. The hormones increase myocardial inotropy and the heart rate and cause the dilation of the peripheral arteries to elevate the cardiac output. Chlamydia pneumonia, Helicobacter pylori, and Influenza virus infections show a greater incidence in winter time as well. Furthermore, respiratory-infection-causing agents play a significant role in the onset of atherosclerosis. Infectious states accompanied by tachycardia and increased cardiac output can cause increased stress on atheromatous plaque^22^. In the case of vulnerable plaque, this can result in an acute cardiovascular incident.

Based on our results, the association between cold weather and ACVD admissions tends to be stronger in elderly patients. However, this is descriptive data that is not specifically related to the primary aim of our study; it is highly possible that the elderly population is more vulnerable to having ACVDs during cold atmospheric conditions. This finding is consistent with a recent study, which demonstrated that the greatest number of hospital admissions for ACVDs concerning the elderly occurred in the cold season^23^. Due to lower thermoregulatory responses, the elderly population has a more pronounced vulnerability to cold atmospheric conditions.

Few studies have investigated an association between warm weather and CVDs^24,25^. In contrast, we observed a lower number of ACVD hospitalisations during the warm season. We did not detect any relationship between warm temperatures and ACVDs although we observed high hospitalisation counts on days with an average temperature of ≥30 °C and high humidity, suggesting an interaction between these two factors.

Our findings are consistent with previous studies that suggested that ACVDs were significantly associated with temperature changes^26,27^. Most studies have shown a greater incidence of ACVDs associated with only a temperature factor. Our study, in contrast, has shown complex, interaction-based atmospheric parameter effects through simultaneous temperature and humidity fluctuations. We found a higher number of ACVD hospitalisations during those atmospheric conditions, when the daily difference in temperature was 5 °C higher and the humidity was 40% higher in the same day. In the case of high humidity, drops of moisture can create a surface of condensation when the vapour is inhaled. This, in turn, triggers a cascade of coagulation and raises the risk of increased onset of thromboembolism. During those days when the temperature change was equal to or more than 5 °C, the number of ACVD hospitalisations increased by 14.0%, whereas it rose even more, by 29.0%, during the days when fluctuations equal to or more than 5 °C occurred along with a 40% or more change in humidity. It could be hypothesized that these results might be due to the fact that the atmospheric parameters have complex and not merely individual effects on the development of ACVDs.

The cardiovascular effects of atmospheric parameters aggregate. A temperature fluctuation poses a higher risk of developing ACVDs if a humidity fluctuation also occurs than without the latter condition’s co-occurrence. We observed that a wind velocity over 2 m/s, both in the cold and hot seasons, enhanced the cardiovascular effects of temperature and humidity interactions when the temperature and humidity fluctuations were ≥5 °C and 40%, respectively. During these specific days, the daily hospitalization rates of ACVDs were higher. The human body adapts to weather changes, but, in some individuals, if the adaptation reaches a certain threshold, the ability to adapt is exhausted, and pathological processes start. The adaptation difficulties are more frequent when more atmospheric parameter changes occur at the same time.

Our study confirms that there is a significant association between atmospheric changes, interactions, and conditions such as hyperlipidaemia, diabetes, and previous CVD history. Based on our research, people possessing these CRFs have a higher risk of cardiovascular morbidity during specific atmospheric conditions due to the fact that they have stronger atmospheric vulnerability. Among vulnerable people, these atmospheric conditions could be defined as cardiovascular trigger factors with pathophysiological changes that increase the onset of ACVDs directly. In Europe, 50% of the population shows meteorological vulnerability. People with meteorosensitivity can be distinguished from those with meteoropathy; meteorosensitive individuals are those who have a biological tendency to perceive changes in atmospheric parameters, whereas people with meteoropathy are individuals who respond to changes in meteorological conditions with a disease or with worsening of an existing disease^28^.

Previous studies reported that there is an association between atmospheric pressure and ACVDs^29^.

In contrast with these results, we did not observe any cardiovascular effects of atmospheric pressure. Also examining the cardiovascular effects of atmospheric parameters on different ACVDs, relative risks for atmospheric effects were not significantly different across patient groups.

There are some potential limitations to this study. Our study used available outdoor atmospheric parameter data to detect population exposure to climatic variables, and data concerning indoor atmospheric conditions were not available. Another aspect that must be considered is the fact that we did not consider certain risk factors, such as socioeconomic status and air pollution.

Our results highlight the complex association between atmospheric parameters and ACVDs. Atmospheric effects could be considered when scheduling elective surgeries, which could reduce the risk of developing postoperative complications. In patients with other pre-existing CRFs, the initiation of preventive pharmacotherapy could be considered regarding atmospheric conditions that pose a risk. Based on the principles of treatment via medication, the timely delivery of the medication is a prerequisite for optimal treatment. In view of this fact, the administration and a possible up-titration of medicines affecting the cardiovascular system are recommended before the onset of atmospheric effects. Furthermore, individuals with meteorological vulnerability should reduce their physical activity during adverse atmospheric conditions. In order to protect against the cold, blood vessel exercise with cold water should be used as a preventive activity to achieve better adaptive abilities.

Moreover, patients with a high risk for cardiovascular diseases may not be prepared to protect themselves against extreme weather conditions that are not typical of their given region. Within the framework of a campaign in England between 1986 and 1987, staying indoors and avoiding outdoor environments were recommended on extremely cold days. As a result, in the given year, there were 30,000 fewer winter deaths than expected^30^. Great importance should be placed on biometeorological forecasts and early warning systems. Biometeorological forecast information should also be available in hospitals, as this would help prepare for adverse atmospheric effects. It is important that the use of a suitable biometeorological forecast system, one that is accessible to the whole population, becomes widespread.

In conclusion, according to our results, the joint effects of wind, temperature, and humidity fluctuations may play an important role in the pathogenesis of ACVDs. Based on our research, patients with hyperlipidaemia, diabetes, and/or previous CVD history have a higher risk of developing ACVDs under the negative effects of the atmospheric parameter conditions. Our previously suggested preventive strategies concerning the negative effects of atmospheric parameters may be more effective when the focus is placed on their complex, interaction-based, joint effects. The importance of our findings is emphasized in the context of extreme atmospheric conditions and changes becoming more likely in the future as a result of climate change. Our findings indicate that population atmospheric vulnerability can be reduced by enhancing the effectiveness of the current public health strategies and innovating future cardiovascular preventive measurements.

## Methods

### Geographical area, atmospheric parameters, and the hospital sampling framework

Retrospectively, we analysed the number of daily hospital admissions due to ACVDs (n = 6499) in a single centre with an intensive Cardiovascular Care Unit during a period of 5 years. Our research project was conducted in Budapest at the Department of Vascular Surgery of Semmelweis University. Budapest, the capital of Hungary, is located in Central Europe in the Carpathian Basin (geographic location coordinates: 47°29′54″ North and 19°02′27″ East). Hungary has a continental climate with several extreme atmospheric situations and an annual mean temperature of between 10 and 11 °C. There is a significant temperature fluctuation between the seasons. The winters are long and cold, and the summer is characterized by warm and dry weather.

The study population was comprised only of those residents with ACVDs from Budapest who were admitted to Semmelweis University’s Heart and Vascular Centre from 2009–2013. We obtained the daily number of hospitalisations using information from the Medsolution IT program database. The ACVDs investigated in our study were determined on the basis of the International Classification of Diseases (ICD). Our study included ACVDs registered in the clinical database, including acute myocardial infarction (I21), pulmonary embolism (I26), dissection of the aorta (I71.0), aortic aneurysm rupture (I71.1/I71.3/I71.5/I71.8), arterial embolism and thrombosis (I74), portal vein embolism and venous thrombosis (I82).

The meteorological approach assumes that, in the same geographical area, all individuals experience the same climatic exposure. Data concerning atmospheric parameters, measured at the weather monitoring station, were obtained from the examined Budapest region by the National Meteorological Service database. Measurements from the meteorological service provided values for each parameter: minimum and maximum temperature (degrees Celsius); minimum and maximum relative humidity (percent); minimum and maximum atmospheric pressure (hPa); and wind speed (m/s). Measurements of atmospheric data were obtained for the day examined. A separate database was created based on the patients’ demographic characteristic (i.e., age) and other major CRFs (e.g., hypertonia, diabetes, hyperlipidaemia, smoking, alcohol, previous CVDs, family history) in order to identify the effects of the atmospheric parameters on major CRFs.

### Statistical analysis

The collected data was subject to descriptive analyses, presenting the average daily hospitalization counts by atmospheric and, to some extent, by demographic parameters as well as by their interactions. Multivariate analyses were carried out in a time-series Poisson-regression framework to explore the adjusted effects of each atmospheric parameter on the ACVD hospitalisation count, allowing for adjustment of the mean and, if deemed relevant, in-day volatility of all measured atmospheric factors. As the present analysis aimed to explore the etiological factors of ACVD hospitalisation counts, the temperature, pressure, humidity, wind velocity, seasonality, and their relevant interactions were included as predictors in the full model. To evaluate the heterogeneity in the associations between hospitalisation counts and atmospheric parameters, the full model was fitted to morbidity subsamples of the complete population. Relative risk estimates were also obtained for all four patient groups admitted with different types of cardiovascular events using the same time-series Poisson regression model. Relying on the asymptotic normality of the maximum likelihood estimates for the model parameters, the Welch corrected F-test p-values showed that that relative risks for atmospheric effects were not significantly different across patient groups.

The statistical analyses were carried out using version 3.4.3 of the R statistical software and the tscount package^31^. Missing data points were omitted from the analyses; the level of statistical significance was set at 5%. The study was approved by the Data Protection Officer of Semmelweis University, according to 21. § (1) from the Hungarian Act CLIV of 1997 on public health.

## Data Availability

The datasets generated and analyzed during the current study are available from the corresponding author on reasonable request.
